# Exploring Distinct Second-Order Data Approaches for Thiamine Quantification via Carbon Dot/Silver Nanoparticle FRET Reversion

**DOI:** 10.3390/bios14120604

**Published:** 2024-12-10

**Authors:** Rafael C. Castro, Ricardo N. M. J. Páscoa, M. Lúcia M. F. S. Saraiva, João L. M. Santos, David S. M. Ribeiro

**Affiliations:** LAQV, REQUIMTE, Department of Chemical Sciences, Laboratory of Applied Chemistry, Faculty of Pharmacy, University of Porto, Rua de Jorge Viterbo Ferreira n° 228, 4050-313 Porto, Portugal; rafael.castro.cl@hotmail.com (R.C.C.); lsaraiva@ff.up.pt (M.L.M.F.S.S.); joaolms@ff.up.pt (J.L.M.S.)

**Keywords:** carbon dots, Förster resonance energy transfer, silver nanoparticles, chemometric analysis, excitation–emission matrices, thiamine

## Abstract

Accurate and selective monitoring of thiamine levels in multivitamin supplements is essential for preventing deficiencies and ensuring product quality. To achieve this, a Förster resonance energy transfer (FRET) system using carbon dots (CDs) as energy donors and citrate-stabilized silver nanoparticles (AgNPs) as energy acceptors was developed. The aqueous synthesis of AgNPs using microwave irradiation was optimized to obtain efficient plasmonic nanoparticles for FRET applications, targeting maximal absorbance intensity, stability, and wavelength alignment. Using a central composite orthogonal design (CCOD), the optimal conditions were identified as a 12.5 min microwave reaction time, a Ag molar ratio of 0.72, and a pH of 8.28. The FRET sensing scheme was applied for thiamine determination, where the vitamin’s presence impaired the FRET process, restoring CDs’ photoluminescence (PL) emission in a concentration-dependent manner. To mitigate interference from other vitamins, PL kinetic data and excitation–emission matrix (EEM) data were analyzed using unfolded partial least-squares (U-PLS) with the subsequent application of the residual bilinearization technique (RBL), achieving high sensitivity and specificity for thiamine detection. This method demonstrated its accuracy and robustness by attaining a determination coefficient (R^2^) of 0.952 and a relative error of prediction (REP%) of 11%. This novel method offers highly sensitive and interference-free thiamine detection, with significant potential for a wide range of analytical applications.

## 1. Introduction

Thiamine, also known as vitamin B1, is an indispensable water-soluble micronutrient naturally found in some foodstuffs [1,2,3]. It is indispensable for numerous oxidation–reduction processes that facilitate the metabolism of glucose and branched-chain amino acids. Thiamine is vital for the oxidative decarboxylation necessary for adenosine triphosphate (ATP) synthesis in the Krebs cycle. It also plays a significant role in the pentose phosphate pathway, where it provides nicotinamide adenine dinucleotide phosphate (NADPH) and pentose phosphates [1]. Despite its presence in many foods, thiamine deficiency can occur due to the consumption of thiamine-poor foods or due to degradation during cooking and/or pasteurization [2]. Due to the body’s limited thiamine stores, deficiency can develop rapidly, leading to conditions such as Wernicke’s encephalopathy, peripheral neuropathy, congestive heart failure, gastrointestinal beriberi, and Korsakoff’s syndrome. This deficiency can also cause complications in critically ill patients, including lactic acidosis, delirium, and gastrointestinal dysfunction [1,2,3]. In cases of vitamin deficiency, supplementation with multivitamins can effectively restore adequate levels of this nutrient. Determining thiamine levels in multivitamin supplements is crucial for preventing deficiencies, ensuring product quality and safety, maintaining efficacy, complying with regulations, and monitoring population nutrition.

Over the past decade, significant efforts have been made to develop analytical methods for the rapid determination of thiamine [4]. Separation techniques, such as capillary electrophoresis [5,6] and high-performance liquid chromatography (HPLC) [7,8,9] have been used as robust approaches. Advances in these methods, particularly in extraction procedures, have reduced sample interference and lowered detection limits, enhancing their applicability in complex matrices. Moreover, these techniques rely on sophisticated and costly equipment, necessitating specialized laboratory facilities, and often involve extensive sample preparation. These methods require highly trained personnel and the associated procedures are labor-intensive and time-consuming, making them less suitable for rapid screening applications.

Additionally, derivatization reactions, which are widely used in thiamine analysis, convert thiamine into thiochrome derivatives through alkaline oxidation, employing reagents like potassium ferricyanide, cyanogen bromide, or mercuric chloride. However, these reagents are highly toxic and pose significant environmental and safety concerns, requiring careful handling [4,10].

To address these limitations, optical methodologies, especially those involving fluorescent nanoparticles, have emerged as attractive alternatives due to their simplicity, reduced need for extensive sample preparation, and potential for rapid and cost-effective vitamin monitoring in commercially available samples [11]. Various approaches can be explored, such as fluorescence quenching sensing schemes [12], time-resolved fluorescence [13], Fluorescence Lifetime Imaging Microscopy (FLIM) [14] and Förster Resonance Energy Transfer (FRET)-based nanoprobes [15].

FRET is an energy transfer mechanism that occurs efficiently without radiation, involving the transfer of energy from an excited donor fluorophore to a ground-state acceptor chromophore via short-range resonant dipole–dipole interactions [16]. This phenomenon happens when the donor’s emission spectrum overlaps with the acceptor’s excitation spectrum, allowing energy transfer over a distance typically less than 10 nm [17]. The efficiency of FRET is strongly influenced by both the distance between the donor and acceptor and the alignment of their dipole moments, rendering it a valuable technique for investigating molecular interactions, conformational dynamics, and binding events at the nanoscale [16,17].

FRET has gained significant attention as a versatile analytical tool in various fields [18], including biophysical studies, deoxyribonucleic acid (DNA) detection, enzyme assays, and bioimaging [19]. Its applications extend to the sensing and quantification of diverse analytes, including toxic substances [20], pharmaceuticals [21], and biomolecules [22]. The exploration of various nanomaterial-based FRET sensing schemes has been particularly noteworthy, owing to their superior photophysical characteristics, namely tunable emission spectra, photostability, and high brightness [16]. In these systems, quantum dots (QDs) or carbon dots (CDs) have been utilized as substitute energy donors rather than fluorescent dyes. CDs have emerged as an attractive alternative to traditional inorganic QDs due to their distinctive chemical and physical characteristics, including low toxicity, ease of synthesis, environmental friendliness, and excellent biocompatibility [23]. CDs possess a combination of quantum dot-like properties and molecular characteristics, which include customizable fluorescence, high chemical stability, and straightforward surface modification [23]. These features make CDs highly suitable for fluorescence-based sensing applications, further broadening the potential of FRET-based technologies.

In parallel, noble metal nanoparticles, such as silver (AgNPs) and gold (AuNPs) can serve as highly effective acceptors for various fluorophores owing to their high molar attenuation coefficients and wide absorption bands [24]. In particular, AgNPs have been explored for their exceptional physicochemical properties, which include ease of functionalization or conjugation with several kinds of ligands, high surface-to-volume ratio, excellent surface plasmon resonance, photostability, size and shape tunability, and quenching capability [25]. However, traditional methods of synthesizing AgNPs often involve toxic and expensive chemicals. Thus, there is a growing interest in developing cost-effective and environmentally friendly synthesis methods that utilize dual-functional compounds acting as stabilizing and reducing agents [23].

In the last few years, FRET systems utilizing CDs and AgNPs have been reported for the detection of various analytes [24,26,27,28]. Despite the advantages, several challenges remain in the widespread application of FRET-based sensing schemes, particularly issues related to stability, signal overlap, and interferences [19,26].

In this sense, chemometric analysis can be used as a powerful solution to address interference issues in FRET-based sensing schemes within chemical analysis. By utilizing PL data analysis with sophisticated chemometric techniques, it becomes possible to effectively comprehend the relationships among samples and analyzed variables, thereby facilitating the determination of one or more analytes in samples with complex matrices [29,30,31]. Analyzing second-order data using suitable chemometric models, such as unfolded partial least-squares (U-PLS) with subsequent the application of the residual bilinearization technique (RBL), enables a second-order advantage [32,33,34]. This advantage facilitates the accurate quantification of analytes, even when uncalibrated and unknown interfering species are present [29]. By using PL kinetic data or excitation–emission matrices processed through chemometric methodologies, researchers can circumvent the potential interference of uncalibrated species, ensuring reliable analytical outcomes.

The main objective of this work was to investigate a FRET sensing system utilizing CDs and AgNPs for the quantification of thiamine in multivitamin dietary supplements. The interaction process between the citrate-stabilized acceptor AgNPs (acceptor) and the CDs (donor) resulted in significant inhibition of the CDs’ photoluminescence (PL) emission. However, the addition of thiamine inhibited the FRET process, thereby restoring the emission effectiveness of the CDs. The PL enhancement was found to be concentration-dependent concerning thiamine. To circumvent uncalibrated interferences from other vitamins in the sample, PL kinetic data and excitation–emission matrices were collected and processed by U-PLS, leveraging the benefits of the second-order advantage. Additionally, the synthesis of AgNPs in an aqueous medium using microwave irradiation was extensively optimized to produce efficient plasmonic nanoparticles for the effective performance of the FRET system with CDs.

## 2. Experimental Methods

### 2.1. Solutions and Reagents

The standards and solutions in this work were prepared utilizing analytical reagent-grade chemicals and requiring no pre-treatment. The water used in the preparation process was purified using a Milli-Q system, assuring a conductivity below 0.1 µS cm^−^^1^.

Silver nanoparticles were synthesized using a microwave-assisted aqueous synthesis route. First, 7.17 mL of a 1 mmol L^−^^1^ silver nitrate (AgNO_3_, Sigma-Aldrich^®^, ≥99%, St. Louis, MO, USA) solution was mixed with 12.83 mL of a 1 mmol L^−^^1^ sodium citrate tribasic dihydrate (HOC(COONa)(CH_2_COONa)_2_.H_2_O, Sigma-Aldrich^®^, ≥99%, St. Louis, MO, USA) solution. The pH of the resulting mixture was then adjusted to 8.28. Finally, the solution was irradiated in a microwave synthesizer for 12.5 min at 100 °C.

CDs were prepared according to the synthetic route outlined by Castro et al. [35,36,37]. Summarily, a mixture was prepared through the dissolution of 0.5 g of citric acid (HOC(COOH)(CH_2_COOH)_2_, Sigma-Aldrich^®^, 99%, St. Louis, MO, USA) and 0.3 mL of ethylenediamine (NH_2_CH_2_CH_2_NH_2_, Sigma-Aldrich^®^, ≥99%, St. Louis, MO, USA) in deionized water, resulting in a citric acid concentration of 10% (*m*/*v*). The pH of this solution was then modified to 3.8 using a 1 mol L^−1^ HCl solution. It was subsequently heated to 260 °C over five hours using a 40 mL Teflon-lined autoclave. After completing the heating process, the mixture was cooled to room temperature. The final product was dialyzed in ultrapure water over five days via a Spectra/Por 6 dialysis membrane with a 1000 molecular weight cut-off (Spectrum Labs™).

The thiamine stock solution was made through the dissolution of 29.5 mg of the thiamine hydrochloride (C_12_H_17_ClN_4_OS.HCl, Sigma-Aldrich^®^, ≥99%, St. Louis, MO, USA) in 35 mL of deionized water.

A commercially available dietary supplement that contains 15 mg of thiamine per tablet was used to prepare the sample solutions. This formulation also includes other vitamins, such as riboflavin, nicotinamide, calcium pantothenate, pyridoxine hydrochloride, biotin, and vitamin B12. To prepare the sample solutions, a suitable quantity of the powdered tablet was measured and then dissolved in deionized water. The mixture was shaken using mechanical stirring for over 30 min to facilitate effective drug extraction. The solution was then filtered into a volumetric flask, and deionized water was added to achieve the final volume.

### 2.2. Instrumentation

Fluorescence spectra were acquired using a PerkinElmer FL-8500 spectrofluorometer (Waltham, MA, USA). The fluorescence lifetime was determined with a DeltaFlexTM TCSPC lifetime spectrofluorometer (Horiba Scientific, Kyoto, Japan).

The synthesis of AgNPs was performed using a CEM Discover SP^®^ Microwave system, equipped with an automated pressure control and sensing system (ActiVent™), an integrated infrared (IR) sensor, and an active cooling system (PowerMAX™). All processes were supervised via a computer interface utilizing Synergy™ software version 1.58 (Matthews, NC, USA). The measurements of pH were performed with a sensION+ pH31 GLP Laboratory pH meter.

A NanoSight NS300 system (Malvern Technologies, Malvern, UK) equipped with a 488 nm blue laser and a high-sensitivity CMOS camera was used to conduct Nanoparticle Tracking Analysis (NTA) on the citrate-stabilized silver nanoparticles (AgNPs), enabling the assessment of their size distribution. To determine the nanoparticles’ average size at room temperature, five consecutive 20 s video recordings were captured. The data were analyzed using NTA 3.4.4 software, applying a detection threshold of 5.

### 2.3. Fluorometric Assay

For the analytical assay preparation, a 1:1500 dilution of the as-prepared solution of CDs was achieved by mixing 100 μL of the crude solution with Milli-Q water, resulting in a volume of 150 mL. Following this, 20 μL of the AgNP solution, along with the required amounts of thiamine solution and deionized water, were introduced into a quartz cuvette with a 10 mm optical path length. Subsequently, 50 μL of the CD intermediate solution was introduced, resulting in a volume of 2 mL.

For the kinetic assay, emission spectra were collected using an excitation wavelength of 351 nm, with slit widths of 5.0 nm for both emission and excitation. Measurements were conducted every minute for a duration of up to 10 min, covering an emission wavelength interval from 400 to 550 nm. For the excitation–emission matrix (EEM) analysis, spectra were obtained with excitation wavelengths varying from 300 to 460 nm, in 3 nm steps, and emission wavelengths varying from 400 to 550 nm in 0.1 nm steps. For both emission and excitation, slit widths of 5 nm were used.

### 2.4. Experimental Design for the Optimization of the AgNP Synthesis Process

A central composite orthogonal design (CCOD) was used for the optimization of the AgNP synthesis using Modde software version 13.0.2.34314 (Umetrics, Malmo, Sweden). Three input factors were considered: microwave (MW) reaction time (from 12.5 to 20.0 min), Ag–citrate molar ratio (from 0.5 to 1.25), and pH (from 7.0 to 8.5). The number of experimental runs was established based on the number of input factors (*k* = 3) using the formula 2*k* + 2*k* + *n*, where *n* is the number of center points. A total of 17 experiments (Table S1—Supplementary Material) were conducted to determine the model’s coefficients (Table S2—Supplementary Material). This design includes a two-level full factorial design (2^3^ = 8), six axial or star points (2 × 3) and three center points. Considering the inclusion of three center points in the CCOD, the value of the axial distance or the star arm is 1.353.

Four distinct output parameters were assessed to determine the quality of the synthesized AgNPs: absorbance intensity at 436 nm, stability of optical properties, wavelength of maximum absorption, and absorbance intensity at the maximum absorption wavelength. The composition of the experiment in terms of the input factors, as well as the results obtained for the output quality properties, is shown in Supplementary Material, Table S1.

### 2.5. Chemometric Analysis

The acquired fluorescence data were analyzed using U-PLS [38] with subsequent application of residual bilinearization technique (RBL) to quantify thiamine. The fluorescence data were acquired using two different approaches: kinetic emission fluorescence and EEM. Both approaches collect second-order data, which provides the second-order advantage, allowing for the effective determination of samples with uncalibrated interferents without including these interferents in the calibration samples [39]. Each EEM spectrum was acquired varying the excitation from 300 to 360 nm, with 3 nm intervals, and varying the emission from 400 to 550 nm, with 0.1 nm intervals, resulting in 31,521 data points (21 × 1501). Each kinetic fluorescence spectrum was collected within 400 to 550 nm using a 0.1 wavelength interval for emission over a total of 10 min, with data collected every minute, yielding a total of 16,511 data points (1501 × 11). Before U-PLS modelling, all spectra were previously mean-centered.

For the quantification of thiamine using the U-PLS model, 8 samples were employed for calibration, while 4 samples were reserved for validation. The validation samples, derived from a dietary supplement, were diluted to four distinct concentrations.

The U-PLS, using the PLS-1 algorithm, begins by refolding the three-dimensional data into a matrix and selecting the ideal quantity of latent variables (LVs) determined from the calibration samples. The ideal quantity of LVs was calculated through the Haaland and Thomas criterion [40], which identifies the suitable count of LVs as the point where the prediction error sum of squares (PRESS) shows no significant difference from the subsequent latent variable, as indicated by an F-ratio probability of less than 0.75. Once the optimal number of LVs is selected, the validation samples can subsequently be introduced into the model. If the validation samples contain uncalibrated interfering species, the RBL procedure should be applied to ensure accurate quantification. This approach employs singular value decomposition to reduce the residual errors of samples containing interfering species to levels comparable to the noise produced by the spectrofluorometer utilized.

The U-PLS/RBL model was built employing the MVC2 interface [41], which calculated all performance metrics using established equations [42], and was executed in Matlab (R2023a version 0.14.0.2254940, MathWorks, Natick, MA, USA).

## 3. Results and Discussion

### 3.1. AgNPs Synthetic Route

The chemical synthesis method has emerged as the most effective and practical approach for producing AgNPs [25]. Several approaches have been employed for the chemical synthesis of AgNPs, including chemical reduction, microemulsion and electrochemical methods [43,44]. Among these, chemical reduction is the most prevalent technique, typically involving the use of metal-based precursors, capping/stabilizing agents, and reducing agents [25,43]. In this process, chemical-reducing agents like sodium borohydride and sodium citrate facilitate the reduction of Ag^+^ ions to metallic silver, resulting in the formation of oligomeric structures. Capping and stabilizing agents play a crucial role in the chemical synthesis of AgNPs, as they help maintain the stability of the silver nanoparticles and inhibit the aggregation of the newly produced nanoparticles.

So, optimizing the synthetic process of AgNPs is essential to obtain efficient nanoparticles with uniform size and shape. This uniformity is primarily determined by the formation of initial nuclei and their ensuing development [44]. These processes are influenced by reaction conditions, including pH, reaction time and the molar ratio between silver precursor, reducing and capping agents [44]. Capping/stabilizing agents are particularly important because they help control the nanoparticle’s growth, allowing for the production of spherical nanoparticles with consistent diameters [44].

The synthesis of AgNPs by chemical reduction can be performed by microwave-assisted heating, which is considered a more efficient method for producing nanoparticles with a higher degree of crystallization and narrower size distribution [25]. The main advantage of microwave-assisted heating over conventional methods is that it provides rapid and uniform heating without needing high temperatures or pressures. Additionally, nucleation and growth processes can be more easily controlled to produce nanoparticles with the required shape or size.

Considering the abovementioned reasons, one objective of this study was the optimization of the aqueous synthesis of AgNPs to produce efficient nanoparticles with the desired optical properties for use in an acceptor–donor FRET assembly with CDs. The synthetic approach involved a one-step microwave-assisted technique that utilized citrate as both a reducing agent and a stabilizer.

#### Optimization of the Synthesis of Citrate-Stabilized AgNPs via Microwave Irradiation

Initially, preliminary assays were carried out to evaluate how important parameters affect the nanoparticle growth rate, stability and optical parameters, and to establish the appropriate value ranges for each parameter. The optimized experimental conditions included microwave reaction time, pH, and the molar ratio between the silver precursor (AgNO_3_) and the reducing/stabilizing agent (trisodium citrate). During this optimization stage, a univariate method was employed to evaluate the impact of each parameter, aiming to produce nanoparticles with the highest absorbance intensity, an absorption wavelength within the emission range of the carbon dots, and stability over a period of 4 weeks.

**Figure 1:**
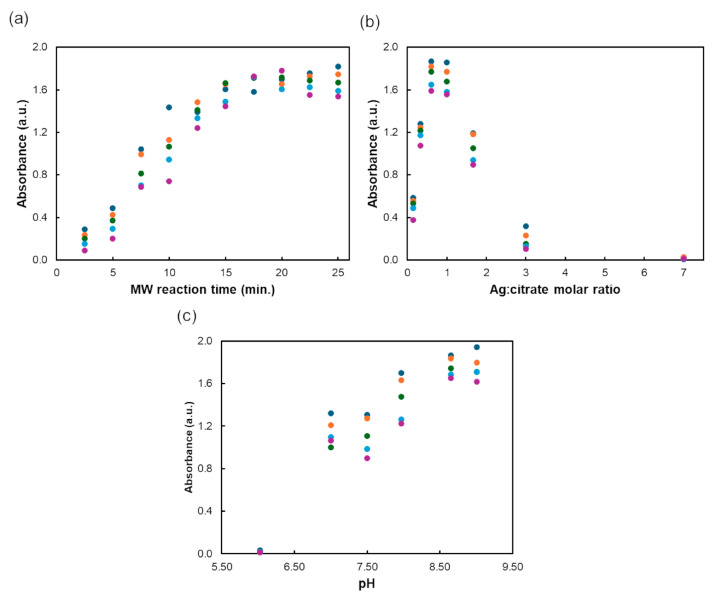
Influence of experimental conditions on the absorbance intensity of AgNPs and their stability over 4 weeks: (**a**) MW reaction time (from 2.5 to 25 min), (**b**) Ag–citrate molar ratio (from 0.14 to 7), and (**c**) pH (from 6 to 9).

The operational parameters of the microwave synthesizer were fixed as follows: temperature at 100 °C, pressure at 200 psi, power at 300 W, and a medium stirring speed. The microwave reaction time was assessed between 2.5 and 25 min, by setting the Ag/citrate molar ratio to 1 and adjusting the pH to 7.0, which corresponds to the pH of the solution resulting from the mixture of the Ag precursor and trisodium citrate at a 1:1 molar ratio. The absorbance spectrum was recorded on the day of synthesis and then weekly for up to 4 weeks. The results demonstrated (Figure 1a) that the absorbance intensity of the AgNPs increased with extended microwave reaction times, up to 12.5 min, after which it stabilized. Additionally, nanoparticles synthesized with reaction times of 10 min or more exhibited higher stability.

The impact of the Ag–citrate molar ratio was studied over a range of values between 0.14 and 7 (specifically, 0.14, 0.33, 0.60, 1.00, 1.67, 3.00 and 7.00), with the MW reaction time fixed at 12.5 min and the pH at 7. The Ag–citrate stoichiometric ratio influenced both the absorbance intensity and the nanoparticle growth rate. As the Ag–citrate molar ratio increased from 0.14 to 7, the wavelength of maximum absorption red-shifted from 431 nm to 508 nm, indicating changes in the nanocrystal growth rate. Additionally, the absorbance intensity (Figure 1b) increased with Ag–citrate molar ratios up to 1 and decreased for higher values.

Finally, the impact of pH on the AgNP synthesis was investigated by fixing the MW reaction time at 12.5 min and the Ag–citrate molar ratio at 1, while varying the pH of the solution from 6.0 to 7.0, 7.5, 8.0, 8.6, and 9.0. The results shown in Figure 1c indicate that increasing the pH led to higher absorbance intensity up to a pH of 8.6, after which the absorbance stabilized. It was also observed that the pH affected the nanocrystal growth rate, as evidenced by the different wavelengths of maximum absorption obtained. However, no clear trend was observed between the change in pH and the obtained wavelengths of maximum absorption.

Based on the information gathered from the preliminary assays, a multivariate experimental design was applied to refine the synthesis conditions, including the MW reaction time, the solution pH, and Ag–citrate molar ratio. The aim was to produce AgNPs with suitable optical properties in terms of absorbance intensity and stability, and to achieve the required wavelength of maximum absorption for use in an acceptor–donor FRET assembly with CDs. Specifically, the absorption band of the AgNPs needs to coincide with the emission band of the CDs for optimal FRET efficiency.

A central composite orthogonal design was applied, as described in Section 2.3. The three input factors and their respective ranges were MW reaction time (from 12.5 to 20.0 min), Ag–citrate molar ratio (from 0.5 to 1.25), and pH (from 7.0 to 8.5). These synthesis conditions were optimized to achieve an optimal compromise between four different output quality properties of AgNPs: absorbance intensity at 436 nm, stability of optical properties, wavelength of maximum absorption, and absorbance intensity at the maximum absorption wavelength. The absorbance intensity at 436 nm was assessed because it corresponds to the maximum emission wavelength of the CDs, which is important for the FRET process efficiency. The stability of the optical properties was quantified by comparing the absorbance intensity at the maximum absorption wavelength on the day of synthesis with the values obtained over the following four weeks. The final stability values were determined by averaging these ratios.

The results of the experimental design used to optimize the AgNPs, based on *p*-values, are shown in the Supplementary Material, Table S2. Regarding output quality parameters such as the absorbance intensity at 436 nm and absorbance intensity at the maximum absorption wavelength, all input factors were highly significant, with pH and Ag–citrate molar ratio demonstrating notable influence. For the stability of the optical properties and the wavelength of maximum absorption, Ag–citrate molar ratio and pH emerged as significant input factors, with Ag–citrate molar ratio having the greatest impact.

The optimal experimental conditions to achieve the highest absorbance intensity at 436 nm, the highest absorbance intensity at the maximum absorption wavelength, stability ratios as close to 1 as possible, and a maximum absorption wavelength as close to 436 nm as possible were an MW reaction time of 12.5 min, a Ag–citrate molar ratio of 0.72, and a pH of 8.28.

### 3.2. Characterization of Carbon Dots and AgNPs

The CDs’ optical properties were assessed via UV–vis spectrophotometry, quantum yield (QY) measurements and both steady-state and time-resolved fluorimetry.

**Figure 2:**
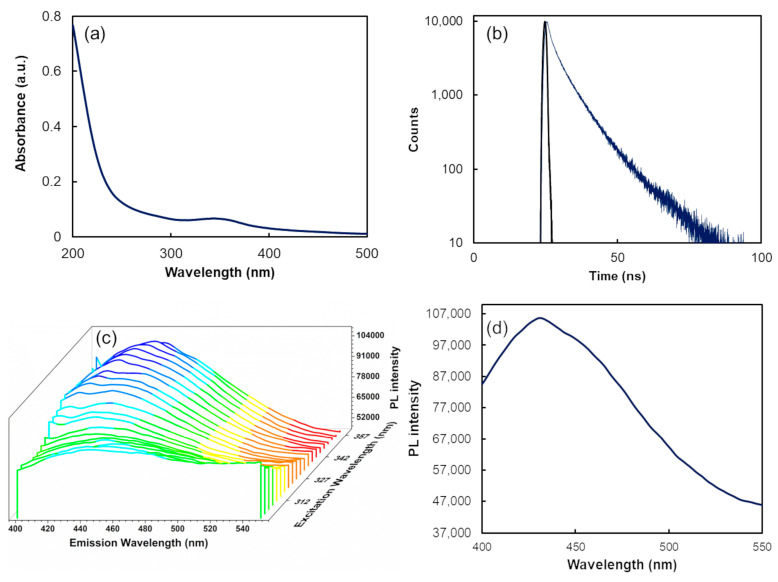
(**a**) CDs’ absorption spectrum. (**b**) PL decay curve of CDs fixing the emission wavelength at 436 nm. (**c**) EEM spectra of CD solution with excitation wavelengths varying from 320 to 390 nm (with increments of 3 nm) and emission wavelengths from 400 to 550 nm. (**d**) CDs’ PL emission spectrum excited at 351 nm.

The UV–visible absorption profile of CDs, shown in Figure 2a, reveals a characteristic absorption band ranging from 320 to 390 nm, with a peak centered at 342 nm, which is attributed to the n–π* transition of the C=O bond [45,46,47,48]. Moreover, the synthesised CDs’ photoluminescence quantum yield (PL QY) was determined to be 20.1 ± 0.3% using an integrating sphere, with the excitation wavelength set at 351 nm.

The CDs’ PL dynamics were analyzed to determine non-radiative and radiative relaxation rates. As depicted in Figure 2b, the emission decay follows a multiexponential pattern, which was modelled using a three-component exponential fit, resulting in a low chi-squared value (χ^2^ = 1.05) and minimal residual deviations. The PL emission comprises three radiative processes: a short-lived component (τ = 1.02 ± 0.01 ns), a medium-lived component (τ = 4.1 ± 0.2 ns), and a long-lived component (τ = 9.51 ± 0.06 ns). The average lifetime, calculated according to Sillen et al. [49], is 8.1 ± 0.1 ns.

The PL spectra were obtained by progressively adjusting the excitation wavelength between 300 and 360 nm in 3 nm steps. As illustrated in Figure 2c, the EEM spectra clearly depend on the excitation wavelength, causing the peak emission wavelengths to transition from 416 nm to 453 nm. Furthermore, the emission band intensity fluctuates in response to the excitation wavelength. The highest peak fluorescence emission intensity occurred at 436 nm with an excitation wavelength of 351 nm (Figure 2d). These findings indicate that the emission characteristics of the CDs are determined by surface states, with variations in surface emission centers resulting in excitation-dependent emission behavior [50].

**Figure 3:**
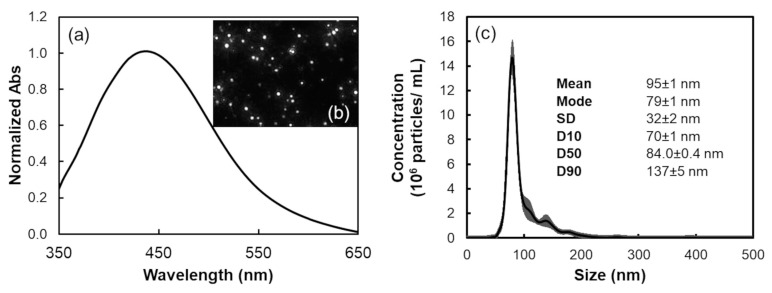
(**a**) Absorption spectrum of the optimized silver nanoparticles; (**b**) caption of video frames showing AgNPs diffracting during measurement; (**c**) size distribution profile AgNPs, derived from the average of five measurements obtained through NTA (video captures).

The characterization of citrate-stabilized AgNPs was conducted using UV–vis spectroscopy and Nanoparticle Tracking Analysis (NTA). NTA was employed to assess the nanoparticles’ size distribution profile. As expected, the AgNPs’ absorption spectrum exhibited a maximum absorption peak at 429 nm (Figure 3a).

Analysis of the light scattered by individual AgNPs in suspension (Figure 3b) revealed that the particles were nearly spherical, as evidenced by the Supplementary Video (Video S1) and the captured frame in Figure 3b. The NTA data enabled the calculation of particle concentration, allowed for the estimation of hydrodynamic diameter, and facilitated the determination of the AgNPs’ size distribution, as shown in Figure 3c. The size distribution profile indicated that the nanoparticles were almost monodisperse, with a mode size of 79 ± 1 nm and an average diameter of 95 ± 1 nm.

Additionally, the AgNPs synthetized exhibited a span of 0.8, a critical parameter in evaluating the particle size distribution [51]. The span is calculated using the formula:

$$Span=\frac{D_{90\;}{-D}_{10}\;}{D_{50}}$$

where $D_{90\;}$ is the diameter under which 90% of the nanoparticles are found, $D_{10}$ is the diameter under which 10% of the nanoparticles are found, and $D_{50\;}$ is the average diameter. A span of 0.8 indicates a relatively narrow size distribution, suggesting that the silver nanoparticles are approximately monodisperse. Moreover, a span of 0.8 reflects a well-controlled production process, essential for quality control and scalability in nanoparticle manufacturing.

### 3.3. Assessment of the Acceptor–Donor FRET System

The subsequent assays aim to evaluate the ability of the synthesized AgNPs to efficiently inhibit the PL emission of CDs through a potential acceptor–donor FRET assembly.

**Figure 4:**
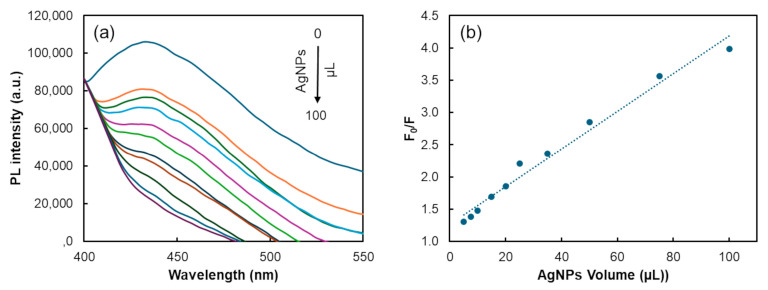
Assessment of the acceptor–donor FRET system. (**a**) PL spectra of CDs with varying volumes of AgNPs; (**b**) Stern–Volmer plot demonstrating the PL intensity ratio of CDs with different volumes of AgNPs.

As depicted in Figure 4, the results showed that the CDs’ PL intensity progressively decreased as larger volumes of AgNPs were added (Figure 4a). This PL inhibition exhibited a direct relationship with the volume of AgNPs added, ranging from 5 to 100 µL (Figure 4b). Additionally, there was no notable change in the maximum emission wavelength, apart from the quenching effect induced by AgNPs.

**Figure 5:**
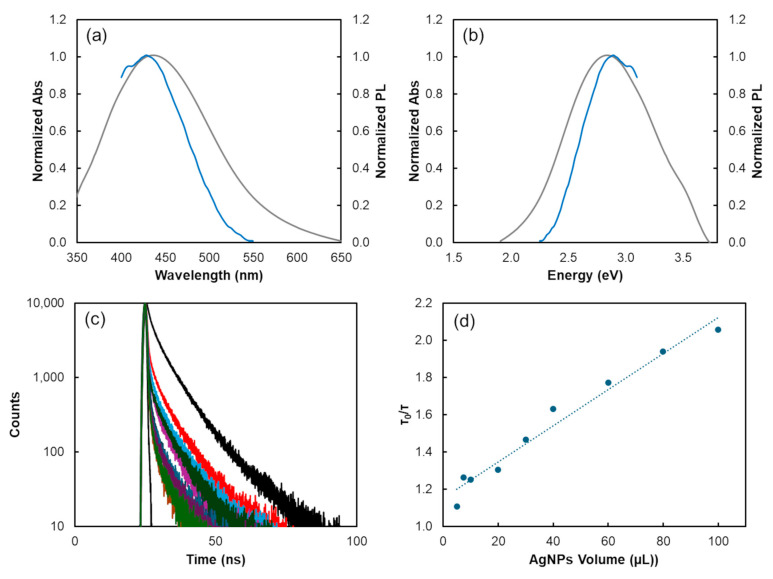
(**a**,**b**) Normalized absorption (grey line) and emission (blue line) spectra of citrate-stabilized AgNPs and CDs, respectively, displayed as a function of wavelength (**a**) and energy (**b**); (**c**) PL decay curves of CDs with varying volumes of AgNPs; (**d**) Stern–Volmer plot exhibiting the PL lifetimes ratio of CDs with different volumes of AgNPs.

In Figure 5a,b, the comparison of the CDs’ emission spectra (maximum emission at 436 nm corresponding to a transition energy of 2.84 eV) with the AgNPs’ absorption spectra (maximum absorption at 429 nm corresponding to a transition energy of 2.89 eV) highlights a significant overlap. This spectral overlap provides the necessary condition for FRET, where the energy from the excited donor (CDs) is transferred to the acceptor (AgNPs). The emission spectrum of the CDs (grey line) overlaps with the absorption spectrum of the AgNPs (blue line), facilitating efficient energy transfer.

Additionally, in FRET processes, the PL lifetime of the donor when the acceptor is present can serve as a distinguishing factor from the Inner Filter Effect (IFE). In FRET, energy transfer from the donor nanoparticle to the acceptor nanoparticle results in a notable decrease in the PL lifetime of the donor. In contrast, under the IFE, there is no energy transfer from the donor to the acceptor nanoparticles, resulting in a relatively stable PL lifetime for the donor molecule. Therefore, it becomes possible to differentiate between the two processes by evaluating the PL lifetime of the donor nanoparticles (CDs) when exposed to varying quantities of AgNPs. A notable reduction in PL lifetime indicates the occurrence of FRET, whereas minimal change suggests the influence of the IFE [52,53].

The PL lifetime of CDs was assessed both without and with increasing concentrations of AgNPs. The introduction of silver nanoparticles led to a reduction in the donor’s PL lifetime (Figure 5c,d), indicating energy transfer from donor to acceptor. This decrease validates the presence of a FRET process.

To maximize the PL intensity inhibition of the CDs while maintaining the visualization of their characteristic PL spectra, a volume of 20 µL of AgNPs was selected for the AgNP–CD FRET assembly.

### 3.4. FRET Reversion Using Thiamine

**Figure 6:**
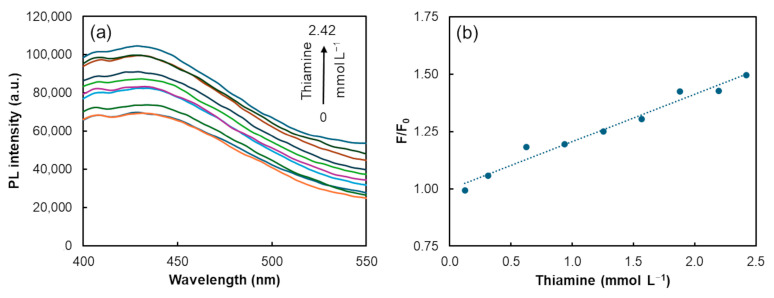
(**a**) PL emission spectra recorded after the addition of thiamine to AgNP–CD FRET assembly; (**b**) linear correlation between the PL signal recovery (*F/F_0_*) and thiamine concentration.

The influence of thiamine on the AgNP–CD FRET assembly was evaluated to study its effect on FRET efficiency. The interaction of thiamine with the FRET assembly was evaluated over a concentration range of up to 2.42 mmol L^−1^, with the results illustrated in Figure 6.

A linear correlation between the PL signal recovery (*F/F_0_*) and the thiamine concentration was observed up to 2.42 mmol L^−1^. The equation for the linear regression is expressed as:

$$\frac{F}{F_{0}}=0.21\left(\pm 0.01\right)\times \left[thiamine\right]+1.00\left(\pm 0.02\right)$$

where *F* and *F*_0_ are the PL intensity of the AgNP–CD FRET assembly without and with thiamine, respectively. The strong linearity is indicated by a correlation coefficient (R) of 0.9740 (*n* = 9).

**Figure 7:**
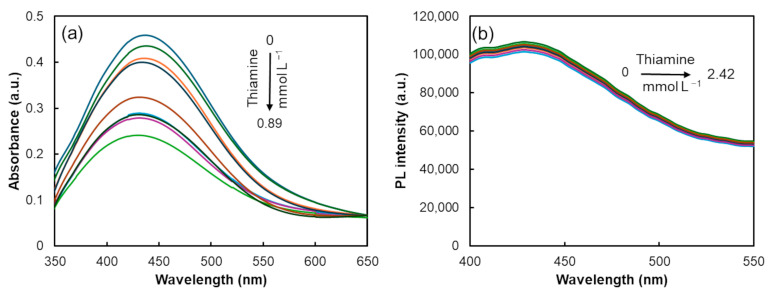
(**a**) Absorption and (**b**) emission spectra of AgNPs and CDs, respectively, upon adding increasing concentrations of thiamine.

Thiamine induced a recovery in PL intensity, attributed to a concentration-dependent reversal of the FRET process. To confirm this hypothesis, the AgNPs’ absorption spectra and the CDs’ emission spectra were recorded both without and with the presence of varying thiamine concentrations. It was verified that higher concentrations of thiamine led to a reduction in the absorption peak of silver nanoparticles (Figure 7a). In contrast, the PL emission of CDs remained practically unchanged with increasing concentrations of the vitamin (Figure 7b). Therefore, it can be concluded that the PL recovery observed in the AgNP–CD FRET assembly was caused by a FRET reversion process.

To address the potential interference from other substances in dietary supplement tablets, two distinct kinds of second-order data were evaluated to identify the most effective method for mitigating the influence of other vitamins. Subsequently, the kinetics and EEM data were studied to further refine the analysis.

### 3.5. Quantification of Thiamine Using U-PLS

#### 3.5.1. Kinetic Approach

**Figure 8:**
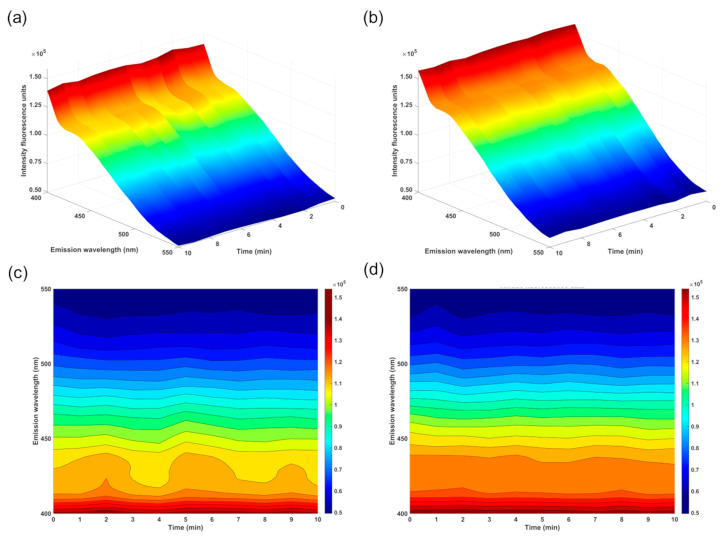
Three-dimensional (**a**,**b**) and two-dimensional (**c**,**d**) representations of PL emission plotted against wavelength and time for the AgNP–CD FRET system (**a**,**c**) and the interaction between the AgNP–CD FRET system and 1.25 mmol L^−1^ of thiamine calibration solution (**b**,**d**).

The study of kinetics was conducted by taking measurements every minute for up to 10 min. Therefore, eight calibration standards with a concentration interval from 0.314 to 2.42 mmol L^−1^ were used. The three-dimensional and two-dimensional kinetic emission spectra of the AgNP–CD FRET assembly in the absence of thiamine (blank) and after the addition of 1.25 mmol L^−1^ of thiamine are revealed in Figure 8.

Both plots reveal that time has no significative influence on the PL signal of the AgNP–CD FRET assembly. The signal remains practically constant for 10 min, likely because the quenching of the absorption intensity of AgNPs upon interaction with thiamine is an instantaneous process. This interaction was studied by evaluating the absorption intensity at 429 nm (maximum absorption wavelength) over the first 10 min (Figure S1—Supplementary Material). After an initial decrease in absorption intensity, the intensity remained unchanged, confirming the minimal changes in the FRET process.

**Figure 9:**
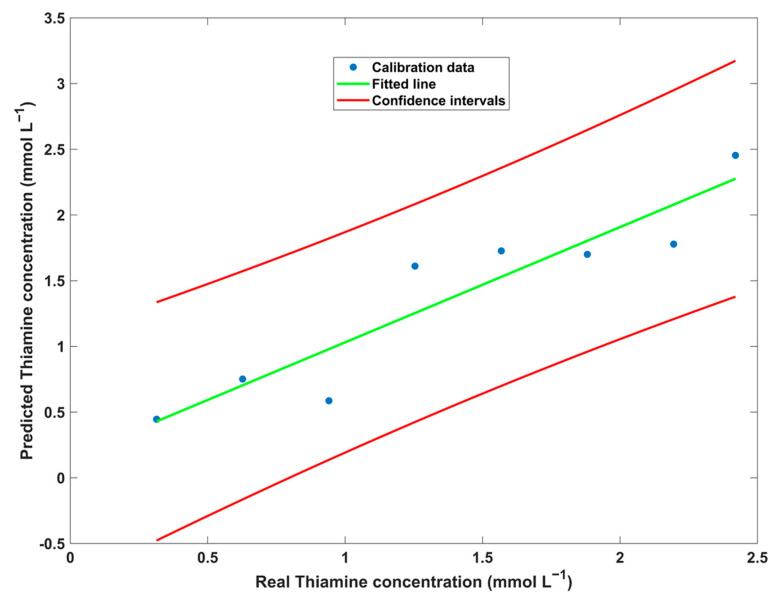
Thiamine calibration curve plot using kinetic fluorescence data and U-PLS.

The developed calibration curve showed some linearity but not as much as intended (Figure 9). This calibration curve was obtained using U-PLS with 1 LV. As can be seen, all values fall within the confidence intervals, considering a confidence level of 95%. A determination coefficient (R^2^) of 0.869 and a root mean square error of cross-validation (RMSECV) of 0.254 were obtained.

After building the calibration model, the validation samples were projected onto this model to assess its accuracy (Table 1).

The results demonstrated that although the developed methodology is sensitive to thiamine, it cannot accurately quantify thiamine in samples containing other vitamins. Indeed, an R^2^_P_ of 0.995 was obtained without the application of RBL, but with a REP value of 36%, indicating a low accuracy of the developed model. The application of 1 and 2 RBL did not yield improvements in the quantification of thiamine using the kinetic PL data. The implementation of RBL was pursued to capitalize on the benefits of second-order advantage, as the sample contains several other ingredients (vitamins) that could interfere with its determination. In terms of LOD and LOQ values, the developed methodology yielded acceptable values. It should be noted that the LOD and LOQ values are obtained after averaging the maximum values of each sample.

#### 3.5.2. EEM Approach

As kinetic studies did not resolve the issue of interfering species in the sample, we investigated the possibility of acquiring EEM matrices to address this challenge. The EEM data for each standard and sample were obtained as described in Section 2.3.

**Figure 10:**
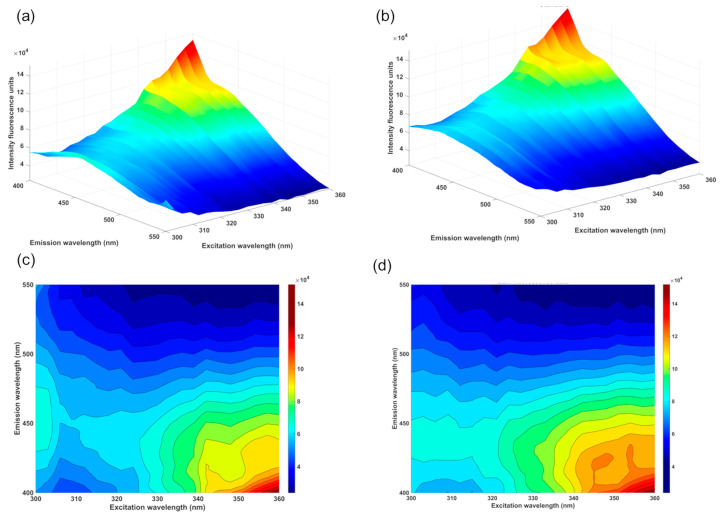
Three-dimensional (**a**,**b**) and two-dimensional (**c**,**d**) plots of the PL emission as a function of excitation wavelength and emission wavelength of AgNP–CD FRET system (**a**,**c**) and upon the interaction between the FRET system and 1.25 mmol L^−1^ of thiamine calibration solution (**b**,**d**).

Therefore, eight distinct standards with thiamine concentrations varying from 0.314 to 2.42 mmol L^−1^ were used for calibration. The three-dimensional and two-dimensional EEM spectra of the AgNP–CD FRET system without the presence of thiamine (blank) and after the addition of 1.25 mmol L^−1^ of thiamine are shown in Figure 10.

**Figure 11:**
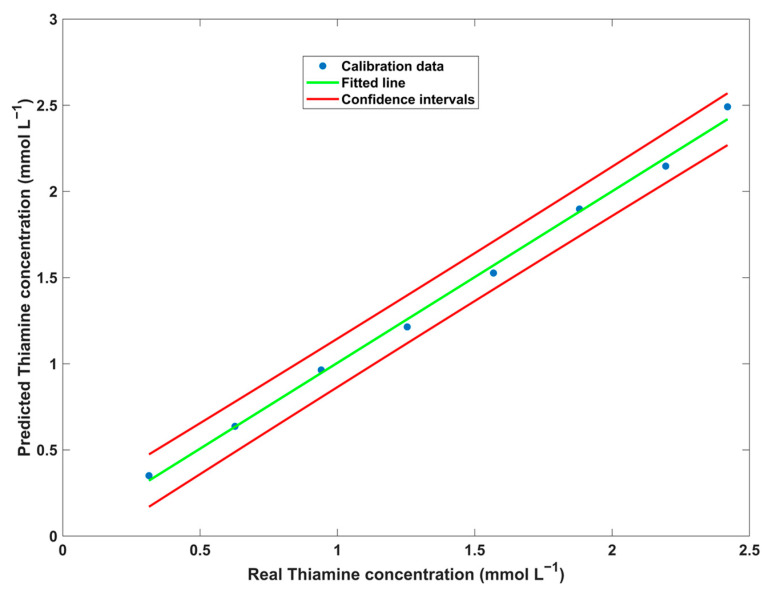
Thiamine calibration curve plot using EEM fluorescence data and U-PLS.

As demonstrated in the characterization of the CDs by the EEM plot, the two-dimensional and three-dimensional EEM plots of the AgNP–CD assembly in the presence of thiamine confirmed the influence of the excitation wavelength on the emission fluorescence signal. In contrast to the kinetic approach, the developed calibration curve using EEM data revealed near-perfect linearity (Figure 11), with all data points closely aligning with the fitted line.

The calibration curve for the EEM data was generated using U-PLS with 1 LV. All data points were found to lie inside the 95% confidence interval. The model achieved a determination coefficient (R^2^) of 0.997 and an RMSECV of 0.040. Following the construction of the calibration model, validation samples were projected onto it to evaluate its accuracy (Table 2).

For the EEM data, the established methodology demonstrated accuracy in quantifying thiamine when employing 1 RBL. The developed model by applying 1 RBL yielded a R^2^_P_ of 0.952 and a REP value of 11%, indicating the effectiveness of the methodology. In this instance, applying RBL to attain the second-order advantage was crucial for accurate predictions. Without applying RBL, the U-PLS model resulted in a REP value of 94%. Despite the presence of several other vitamins in the sample, applying 1 RBL was sufficient to overcome their interference. Regarding LOD and LOQ values, the developed methodology produced acceptable results for both parameters, with values below the concentration of the first calibration standard and within the expected range for commercial samples (15 mg per tablet).

In this context, the developed methodology, based on a FRET reversion process using AgNPs and CDs, proved to be suitable and accurate for the quantification of thiamine in complex matrices, even when several uncalibrated ingredients are present in the validation samples.

## 4. Conclusions

This study successfully optimized the synthesis of citrate-stabilized AgNPs using a microwave-assisted method, achieving the desired optical properties for FRET applications. Optimal synthesis conditions, determined through a CCOD, produced AgNPs with high absorbance intensity at 436 nm and stable optical properties over four weeks. These AgNPs were successfully integrated into a FRET assembly with CDs, demonstrating efficient quenching of CD photoluminescence, which was reversed by thiamine in a concentration-dependent manner, enabling its quantification.

Two methodologies, kinetic and EEM approaches, were employed for selective thiamine quantification in the presence of other vitamins and interfering substances. The kinetic approach was inadequate due to the prompt reversal of FRET by thiamine, yielding a lower determination coefficient (R^2^ = 0.869) and higher relative error of prediction (REP% = 36%), highlighting the limited significance of kinetics in this context. In contrast, the EEM approach, coupled with U-PLS regression and RBL, provided significantly improved accuracy. The calibration model using EEM data and RBL achieved a determination coefficient (R^2^) of 0.952 and a REP% of 11%. The use of RBL was essential, providing the benefits of second-order advantage while effectively reducing interference from other substances in the samples.

The results indicate that the EEM approach is a more robust and accurate method for thiamine quantification compared to the kinetic approach. The developed FRET-based methodology, utilizing the reversal of energy transfer in the presence of thiamine, offers a sensitive and reliable analytical method for detecting thiamine in complex matrices. The use of chemometric models in CD-based FRET systems represents a significant advancement, addressing interference challenges in chemical analysis. This reliable and sustainable approach enhances the accurate determination of analytes in samples containing interfering species, particularly in pharmaceutical and nutritional contexts where accurate quantification of vitamins and other compounds is crucial.

## Figures and Tables

**Table 1:** Results of predicted thiamine concentrations obtained through U-PLS/RBL analysis of validation samples, along with the corresponding figures of merit for the proposed method utilizing kinetic fluorescence data.

Test Sample	[Thiamine] mmol L^−1^			
	Real	Predicted		
		0 RBL	1 RBL	2 RBL
1	1.00	1.53	1.12	0.48
2	1.19	1.72	1.95	1.90
3	1.63	2.20	1.53	1.14
4	2.01	2.33	1.19	1.45
**RMSEP (mmol L^−1^)**		0.497	0.563	0.575
**R^2^_P_**		0.955	0.049	0.110
**REP% (%)**		36	40	41
**LOD (mmol L^−1^)**		0.053	0.069	0.078
**LOQ (mmol L^−1^)**		0.160	0.207	0.234

RMSEP—root mean square error of prediction; R^2^_P_—determination coefficient of prediction; REP%—relative error of prediction; LOD—limit of detection; LOQ—limit of quantification.

**Table 2:** Results of predicted thiamine concentrations obtained via U-PLS/RBL for validation samples, along with the corresponding figures of merit for the proposed method utilizing EEM fluorescence data.

Test Sample	[Thiamine] mmol L^−1^			
	Real	Predicted		
		0 RBL	1 RBL	2 RBL
1	1.00	2.45	1.29	1.78
2	1.19	2.66	1.20	1.44
3	1.63	2.87	1.71	1.50
4	2.01	3.08	1.90	2.02
RMSEP (mmol L^−1^)		1.32	0.160	0.413
R^2^_P_		0.989	0.952	0.477
REP% (%)		94	11	29
LOD (mmol L^−1^)		0.028	0.064	0.064
LOQ (mmol L^−1^)		0.085	0.192	1.92

RMSEP—root mean square error of prediction; R^2^_P_—determination coefficient of prediction; REP%—relative error of prediction; LOD—limit of detection; LOQ—limit of quantification.

## Data Availability

The raw data supporting the conclusions of this article will be made available by the authors on request.

## Supplementary Materials

The following supporting information can be downloaded at: https://www.mdpi.com/article/10.3390/bios14120604/s1. Figure S1: Normalized absence intensity of the AgNPs in the absence (blue circle) and in the presence of 0.13 (orange squares) and 0.89 mmol^−1^ (green triangles) of thiamine; Table S1: Experimental design for the optimization of AgNPs synthesis; Table S2: Results of the experimental design for the optimization of AgNPs synthesis in terms of *p*-value; Video S1: NTA analysis of AgNPs over 20 s.
