# Supplementary material for: Exploring Distinct Second-Order Data Approaches for Thiamine Quantification via Carbon Dot/Silver Nanoparticle FRET Reversion
Source: Biosensors (Basel). 2024 Dec 10;14(12):604. doi: 10.3390/bios14120604 (PMC11674425; doi:10.3390/bios14120604)
Supplement: Supplementary file 1 [file biosensors-14-00604-s001.zip › biosensors-3330653-supplementary.pdf]

biosensors
MDPI

Supplementary Material

Exploring Distinct Second-Order Data Approaches for Thiamine Quantification via Carbon Dot/Silver Nanoparticle FRET Reversion

Rafael C. Castro, Ricardo N.M.J. Páscoa *, M. Lúcia M. F. S. Saraiva, João L.M. Santos, David S.M. Ribeiro *

Table S1. Experimental design for the optimization of AgNP synthesis.

Number	Input factors			Output quality properties			
	Time (min)	Molar ratio	pH	Intensity at 436 nm	Stability	Absorption maximum wavelength	Absorption maximum intensity
1	20.0	0.50	6.97	0.593	0.881	455.5	0.622
2	21.5	0.88	7.84	0.792	0.868	449.8	0.808
3	16.4	0.88	6.78	0.295	0.691	478.2	0.334
4	12.5	0.50	6.98	0.588	0.915	456.2	0.619
5	20.0	1.25	6.98	0.095	0.456	520.5	0.108
6	16.2	0.88	8.96	0.765	0.773	446.3	0.772
7	11.0	0.88	7.73	0.966	0.938	453.5	0.991
8	16.4	0.88	7.84	0.909	0.802	448.5	0.922
9	16.2	0.88	7.73	0.721	0.667	460.3	0.749
10	12.5	0.50	8.49	0.891	1.015	421.2	0.937
11	12.5	1.25	7.04	0.478	0.609	473.8	0.507
12	16.2	0.88	7.80	0.929	0.842	451.0	0.949
13	12.5	1.25	8.61	0.816	0.843	460.8	0.845
14	16.4	0.37	7.91	0.728	0.976	411.3	0.916
15	20.0	0.50	8.54	0.941	1.016	421.7	0.984
16	20.0	1.25	8.63	0.547	0.668	475.5	0.579
17	16.4	1.38	7.73	0.169	0.589	490.8	0.184

Table S2. Results of the experimental design for the optimization of AgNP synthesis in terms of *p*-value.

	p-values			
	**Intensity at 436 nm**	**Stability**	**Absorption maximum wavelength**	**Absorption maximum intensity**
**Time (min)**	0.043	0.083*	0.053*	0.045
**Molar ratio**	0.001	0.000	0.000	0.001
**pH**	0.000	0.006	0.001	0.000
**Time × Time**	0.108*	0.032	0.851*	0.197*
**Molar ratio × molar ratio**	0.003	0.921*	0.840*	0.012
**pH × pH**	0.012	0.140*	0.053*	0.010
**Time × molar ratio**	0.039	0.135*	0.033	0.043
**Time × pH**	0.684*	0.999*	0.249*	0.655*
**Molar ratio × pH**	0.462*	0.224*	0.710*	0.518*

* not statistically significant.

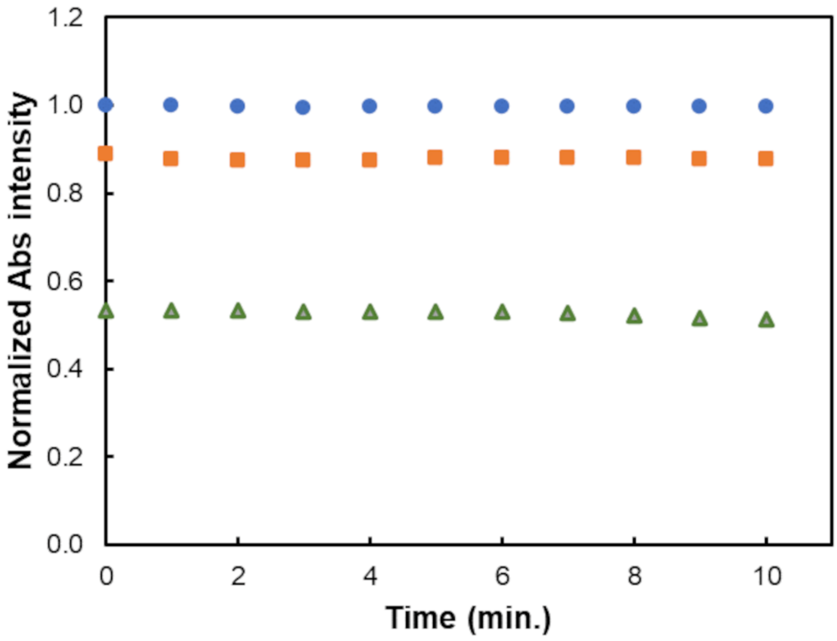

Figure S1. Normalized absence intensity of the AgNPs in the absence (blue circle) and in the presence of 0.13 (orange squares) and 0.89 mmol-1 (green triangles) of thiamine.
